# Direct Determination of Peroxide Explosives on Polycarbazole/Gold Nanoparticle-Modified Glassy Carbon Sensor Electrodes Imprinted for Molecular Recognition of TATP and HMTD

**DOI:** 10.1021/acs.analchem.2c04450

**Published:** 2022-12-06

**Authors:** Şener Sağlam, Ayşem Üzer, Reşat Apak

**Affiliations:** † Engineering Faculty, Chemistry Department, Istanbul University-Cerrahpaşa, Avcilar, 34320Istanbul, Turkey; ‡ Turkish Academy of Sciences (TUBA), Bayraktar Neighborhood, Vedat Dalokay st. No.: 112, Cankaya, 06670Ankara, Turkey

## Abstract

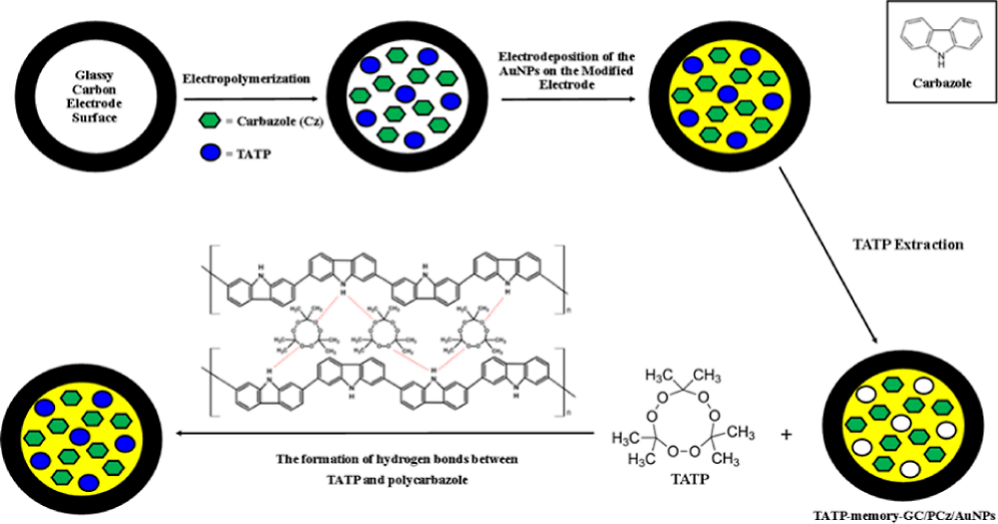

Since peroxide-based explosives (PBEs) lack reactive functional groups, they cannot be determined directly by most detection methods and are often detected indirectly by converting them to H_2_O_2_. However, H_2_O_2_ may originate from many sources, causing false positives in PBE detection. Here, we developed a novel electrochemical sensor for the direct sensitive and selective determination of PBEs such as triacetone triperoxide (TATP) and hexamethylene triperoxide diamine (HMTD) using electrochemical modification of the glassy carbon (GC) electrode with PBE-memory polycarbazole (PCz) films decorated with gold nanoparticles (AuNPs) by cyclic voltammetry (CV). The prepared electrodes were named TATP-memory-GC/PCz/AuNPs (used for TATP determination) and HMTD-memory-GC/PCz/AuNPs (used for HMTD detection). The calibration lines of TATP and HMTD were found in the concentration range of 0.1–1.0 mg L^–1^ using the net current intensities of differential pulse voltammetry (DPV) *versus* analyte concentrations. The limit of detection (LOD) commonly found was 15 μg L^–1^ for TATP and HMTD. The sensor electrodes could separately determine intact TATP and HMTD in the presence of nitro-aromatic, nitramine, and nitrate ester energetic materials. The proposed electrochemical sensing method was not interfered by electroactive substances such as paracetamol, caffeine, acetylsalicylic acid, aspartame, d-glucose, and detergent (containing perborate and percarbonate) used as camouflage materials for PBEs. This is the first molecularly imprinted polymeric electrode for PBEs accomplishing such low LODs, and the DPV method was statistically validated in contaminated clay soil samples against the GC-MS method for TATP and a spectrophotometric method for HMTD using *t*- and *F*-tests.

Peroxide-based explosives (PBEs) cannot be used for industrial and military purposes due to their unstable nature^1,2^ but are popular among terrorist groups because the starting materials used in the synthesis of these compounds can be easily obtained (e.g., hydrogen peroxide from hair dyes and antiseptics and acetone from nail polish removers).^3,4^ PBEs such as triacetone triperoxide (TATP) and hexamethylene triperoxide diamine (HMTD) were first captured by the Israel Police in the late 1970s. The London underground attacks in 2005, in which many people were killed and injured, are well-known incidents related to the use of TATP. The determination of PBEs has gained importance in recent years due to the actions that have had repercussions all over the world,^5^ and this has led researchers to develop fast and reliable detection methods for PBEs.^6,7^

PBEs do not respond to most spectroscopic detection methods because they lack reactive functional groups.^8^ In the spectrophotometric determination of these explosives, it is often necessary to hydrolyze the PBE in medium–strong acid to obtain H_2_O_2_ and neutralize the excess acid. In other words, indirect determination of explosives is made by the analysis of H_2_O_2_ released by the decomposition of PBEs.^9^ Several analytical methods have been developed for the determination of PBEs such as spectroscopy,^10^ fluorometry,^11^ liquid chromatography (LC),^12^ LC coupled with mass spectrometry^13^ or gas chromatography using mass detector (GC–MS),^14^ and thin-layer chromatography.^15^ However, these sophisticated techniques are relatively expensive, laborious, and solvent/time-consuming. As excellent alternatives to these methods, electrochemical techniques can be preferred because of their fast response, easy operation, high sensitivity and selectivity, low cost, and portability.^16^

PBEs can be electrochemically determined using various working electrodes such as glassy carbon electrodes (GCEs), screen-printed electrodes, and carbon fiber electrodes.^5,17^ Moreover, the sensor working electrodes can be formed by modifying their surfaces with different polymeric materials^18^ and nanomaterials (nanotubes and/or nanoparticles)^19^ in order to carry out sensitive and selective detection of PBEs. Molecularly imprinted/memory techniques are sophisticated methods for the development of sensor electrodes which have cavities that can identify a specific molecule in terms of shape, size, and functional group.^20,21^ The potential analyte, which is intended to be determined sensitively and selectively, is used as a template molecule during polymer synthesis. This template molecule is extracted from the matrix medium after polymer synthesis. The major advantages of molecularly imprinted polymers (MIPs) are their high selectivity and affinity to the target molecule.^22−24^

Electrochemical determinations of PBEs are usually performed indirectly, similar to spectroscopic methods. Namely, PBEs are converted to H_2_O_2_ by hydrolysis in an acidic medium, and the resulting H_2_O_2_ (decomposition product) is analyzed electrochemically.^25,26^ However, the direct electrochemical methods for the determination of intact PBEs without degradation into H_2_O_2_ are very valuable and quite few in the literature. In a rare example, Mamo and Gonzalez-Rodriguez improved a MIP-based electrochemical sensor electrode for TATP determination. The surface of the GCE was modified by cyclic voltammetry (CV) using pyrrole as a functional monomer, TATP as a template molecule, and LiClO_4_ as an electrolyte *via* electrochemical polymerization. The determination of TATP using the improved electrode was accomplished with differential pulse voltammetry (DPV) in the concentration range of 82–44300 μg L^–1^, and the limit of detection (LOD) was 26.9 μg L^–1^. In this study, real sample analysis and PBE determination in the presence of H_2_O_2_ were not investigated.^18^ Krivitsky et al. prepared a selective and direct electrochemical vapor detection of HMTD and TATP explosives using an Ag nanoparticle-modified carbon microfiber air-collecting electrode by the DPV method. The linear calibration curves lay in the range of 30–150 mg L^–1^ for TATP and HMTD. However, the indicated method was used for direct analysis at pH 12 where most organic compounds are prone to oxidation. Moreover, the lack of interference from H_2_O_2_ in HMTD/TATP analysis was effective merely within a limited concentration ratio of H_2_O_2_ to PBEs.^17^ Arman et al. proposed an electrochemical sensor for direct analysis of TATP and HMTD using well-dispersed multi-walled carbon nanotubes/polyethyleneimine-modified GCE. This modified sensor electrode responded to intact TATP in a neutral medium. The direct electrochemical reduction of TATP and HMTD was carried out using the DPV method in an 80/20% (v/v) H_2_O–acetone solvent medium within the concentration range of 10–200 mg L^–1^ for TATP and 25–200 mg L^–1^ for HMTD, and the detection limits were 1.5 mg L^–1^ and 3.0 mg L^–1^, respectively. Additionally, common ions, different types of energetic materials such as nitro-aromatic and nitramine-type explosives, and electroactive camouflage materials did not interfere with the proposed method.^27^

In this study, we prepared peroxide-based energetics memory-polycarbazole (PCz) films decorated with gold nanoparticles (AuNPs) for direct, sensitive, and selective determination of TATP and HMTD by the DPV method using cathodic peak current measurement. These sensor electrodes were prepared in two steps. In the first step, the GC electrode surface was modified with the target molecule (template) and the carbazole (Cz) monomer *via* electrochemical polymerization. TATP and HMTD were used as template molecules. In the second step, the surface of the PBE memory-poly(carbazole) electrode was functionalized with AuNPs. The prepared electrodes were named TATP memory-GC/PCz-AuNPs-modified electrode and HMTD memory-GC/PCz-AuNPs-modified electrode. A separate electrode was used for each PBE with special recognition of the intact target analyte. The prepared sensor electrode was characterized using scanning electron microscopy (SEM), impedance measurements, and CV. Moreover, TATP and HMTD were selectively determined in the presence of nitro-aromatic and nitramine-type energetic material mixtures (binary and multicomponent mixtures). Additionally, some electroactive compounds carried as passenger belongings such as caffeine, paracetamol, aspartame, acetylsalicylic acid, detergent (containing perborate and percarbonate), and d-glucose that can be used as possible camouflage materials having similar color and appearance did not interfere with PBE determination. Finally, the developed voltammetric procedure was validated against a literature GC–MS method^28^ for TATP and a spectrophotometric method^9^ for HMTD on contaminated clay soil samples using statistical *t*- and *F*-tests. The developed procedure is important as it is the first interference-free method to directly detect TATP and HMTD with a MIP sensor electrode at a low LOD relevant for testing environmental samples.

## Experimental Section

### Safety Note

Peroxide-based energetics such as TATP and HMTD are highly hazardous materials that can cause spontaneous explosions and violence under friction, impact, and temperature changes. The synthesis of these substances must be carried out by qualified personnel under safety precautions and in small quantities not exceeding 100 mg (*i.e.*, higher amounts increase the risk of spontaneous explosions).^29^ TATP and HMTD were synthesized as described in the literature.^30,31^

### Chemicals

Nitro-based explosive materials such as 2,4,6-trinitrotoluene (TNT), 2,4-dinitrotoluene (DNT), 2,4,6-trinitrophenylmethylnitramine (tetryl), picric acid (PA), octahydro-1,3,5,7-tetranitro-1,3,5,7-tetrazosin (HMX), 1,3,5-trinitroperhydro-1,3,5-triazine (RDX), pentaerythritol tetranitrate (PETN), Comp B composite explosive (containing 60% RDX, 39% TNT, and 1% wax), and Octol composite explosive (containing 70% HMX and 30% TNT) standards were supplied from the Machinery & Chemistry Industries Institution (MKEK) of Turkey from previous projects.

For cleaning the working electrode, an alumina slurry from Baikowski International Corp. (0.05 mm, Baikalox 0.05CR), isopropanol (Sigma-Aldrich), technical ethanol, and acetone (Emboy) were used. The supporting electrolytes were tetraethylammonium perchlorate (TEAP) (used for electropolymerization) (Sigma-Aldrich) and tetrabutylammonium tetrafluoroborate (TBABF_4_) (used for analysis) (Aldrich) for preserving conductivity. For the synthesis of TATP, hydrogen peroxide solution (30%, puriss., stabilized, Sigma) and acetone (Chromasolv, for HPLC, ≥99.8%, Sigma) were used. For the spectrophotometric analysis of HMTD, sodium hydroxide (NaOH) (Merck), hydrochloric acid (HCl) (Sigma), copper (II) chloride (CuCl_2_) (Merck), ammonium acetate (NH_4_Ac) (Merck), and neocuproine (Nc) (2,9-dimethyl-1,10-phenanthroline) (Merck) were used. Carbazole (Cz), also known as 9*H*-carbazole or diphenylenimine (used as a monomer for the polymeric coating of a sensor electrode), together with other chemicals, was purchased from E. Merck (Darmstadt, Germany).

### Solutions

The standard stock solutions of explosives, as well as military-purpose mixed explosives (Comp B and Octol), were prepared in extra pure acetone (Sigma-Aldrich). The monomer solution of carbazole was prepared in extra pure acetonitrile (AcCN). Gold (III) chloride solution (HAuCl_4_ of 99.99% purity on trace metals basis, containing ≤150.0 ppm trace metals, 30% by wt in dilute HCl) was utilized for AuNP modification of the PBE memory-GC/PCz working electrode surface, and the 0.04% (w/v) working solution was prepared from this stock solution. The stock solutions of camouflage materials (caffeine, paracetamol, aspartame, acetylsalicylic acid, detergent, and d-glucose) at a concentration of 1000 mg L^–1^ were prepared in acetone and filtered through Chromafil PET-45/25 (a 0.45 μm filter) (Macherey-Nagel) before measurements.

### Instruments

Voltammetric experiments were carried out with a Metrohm Autolab potentiostat/galvanostat (PGSTAT204, Switzerland) and controlled by Nova 2.1.5 software. In addition, the GC electrode (BASI MF-2012), Ag/AgCl electrode (BASI MF-2052), and platinum (Pt) wire electrode were used as the working electrode, reference electrode, and auxiliary electrode, respectively.

The developed method was validated against GC/MS using a Thermo Scientific Trace gas chromatography coupled with a quadrupole analyzer and a DSQII MS containing electron impact ionization for TATP and a UV–vis spectrophotometer (Shimadzu UV-1800) for HMTD. The surface analysis of the developed MIP sensor electrode was carried out with an FEI Model Quanta 450 FEG Scanning Electron Microscopy (SEM) instrument.

### TATP and HMTD Synthesis

TATP synthesis was achieved by mixing 1.77 mL of acetone with 2.55 mL of 30% hydrogen peroxide at 0 °C under the catalysis of five drops of concentrated sulfuric acid. The obtained product was kept at room temperature and acetone atmosphere for 24 h after synthesis. The liquid part remaining on the white solid was decanted, washed with 100 mL of a 70% water–30% acetone mixture, and filtered through a cellulose acetate filter with the help of a vacuum pomp.^30^ The product was stored in glass storage boxes with a cap at 4 °C in acetone vapor. The synthesized material structure was clarified by the carbon and hydrogen percentages of TATP (an empirical formula C_9_H_18_O_6_, *M*_w_: 224.24 g mol^–1^) using elemental analysis (expected: C 48.6 and H 8.16%; found: C 48.4 and H 8.13%).

HMTD synthesis was performed by mixing 1.4 g of hexamethylenetetramine, 13.5 mL of 30% H_2_O_2_, and 2.1 g of citric acid at 0 °C for 3 h and keeping at room temperature for 2 h. Later, the formed crystals were washed with 50 mL of distilled water and 25 mL of methanol and filtered through a cellulose acetate filter using a vacuum pump. The product was stored in a glass bottle with a cap at 4 °C in acetone vapor. The synthesized material structure was clarified by carbon and nitrogen percentages of HMTD (an empirical formula C_6_H_12_O_6_N_2_, *M*_w_: 208.17 g mol^–1^) using elemental analysis (expected: C 34.6 and N 13.45%; found: C 34.9 and N 13.4%).

### Cleaning Procedure of the Working Electrode

The GCE was cleaned with a suspension of alumina in circular motions for 5 min and then washed with distilled water before modification. Later, the GCE was sonicated in distilled water and a mixture of isopropanol–acetonitrile (1:1, v/v) for 5 min.^32^

### Method Optimization

The experimental parameters such as the selections of the working electrode, solvent, and supporting electrolyte and the concentration of the latter were investigated separately. The characteristic reduction peak potentials of PBEs were obtained using the DPV method with the GCE chosen as the working electrode. The scan rate was selected as 20 mV s^–1^; TBABF_4_ and acetone were used as the supporting electrolyte (with an optimal concentration of 0.025 mol L^–1^) and solvent in this work, respectively.

### Preparation of PBE-MIP Sensor Electrodes

The preparation of the PBE-MIP electrode was carried out in two steps. In the first step, 5 mL of a solution (prepared in AcCN) containing 1.0 × 10^–2^ mol L^–1^ carbazole (Cz) monomer, 100 mg L^–1^ template molecule (TATP for the TATP-MIP sensor electrode and HMTD for the HMTD-MIP sensor electrode), and 0.1 mol L^–1^ TEAP supporting electrolyte were taken into the working cell. The electropolymerization process was carried out using the CV method within the potential range (−1.8 to 1.6 V) at a scanning speed of 20 mV s^–1^ and for five cycles.

In the second step, the prepared electrode was stabilized by polymerizing the remaining monomers, dimers, and oligomers on the surface of the modified working electrode in the supporting electrolyte (without the monomer and the template molecule) with the CV method using the same potential range, scanning speed, and cycle number as in the first step. Finally, the modified GCE was rinsed with AcCN for removing any unbound material from the surface so as to form the PBE-memory-GC/PCz electrode.

### AuNPs—Modification of the Surface of the PBE-MIP Sensor Electrode

AuNPs were accumulated on the PBE-memory-GC/PCz modified electrode surface by using 0.04% (w/v) HAuCl_4_ (2.5 mL) + 0.1 mol L^–1^ H_2_SO_4_ (2.5 mL) solutions by the CV method through electrochemical deposition. The deposition process was carried out at a scan rate of 50 mV s^–1^ within the range of (−0.4 to 0.4 V), and the amount of AuNPs accumulated on the surface was estimated by controlling the number of cycles at an optimal value of 40 cycles.^33^ As a result of this process, the golden-colored electrode was formed and named as the PBE-memory-GC/PCz/AuNPs sensor electrode. The characterization details of the developed sensor electrode such as CV measurements, impedance measurements, and SEM image are given in the Supporting Information (Figures S1–S3).

### Electrochemical Measurements of TATP and HMTD

The working solutions of 0.1–1.0 mg L^–1^ TATP and HMTD were prepared in acetone from the corresponding stock solutions, and 5 mL of solution was transferred to the measurement cell, to which 0.025 mol L^–1^ TBABF_4_ was added as the supporting electrolyte. The modified electrode was cleaned in acetone for 1 min before each electrochemical measurement. By performing this step, any analyte remaining from the previous measurement was removed from the modified electrode surface. The DPV method was used within the potential range of (0.4 V to −1.6 V), and the reduction peak potentials of TATP and HMTD were found. The TATP-memory-GC/PCz/AuNPs for TATP determination and the HMTD-memory-GC/PCz/AuNPs for HMTD determination were used as sensor electrodes.

### Analysis of Synthetic and Real Energetic Material Mixtures

TATP and HMTD solutions at 0.5 mg L^–1^ were separately analyzed in the presence of 10-fold concentrations of nitroaromatic energetic materials (TNT, DNT, tetryl, and PA), nitramine-type energetic materials (RDX and HMX), nitrate ester-type energetic material (PETN), and real energetic mixtures such as Comp B and Octol using the proposed DPV method.

### Assay in the Presence of Electroactive Camouflage Materials

The electroactive camouflage materials having similar color and appearance with the analytes such as paracetamol–caffeine-based analgesic drugs, acetylsalicylic acid, aspartame-based sweeteners, detergent, and sugars were studied. TATP and HMTD solutions at 0.5 mg L^–1^ were determined in the presence of 200-fold (12-fold for caffeine) concentrations of electroactive compounds using the TATP-memory-GC/PCz/AuNPs and HMTD-memory-GC/PCz/AuNPs sensor electrodes by the developed DPV method. Besides these electroactive materials, the analyses of TATP and HMTD were also made in the presence of 3-fold concentrations of H_2_O_2_. The preparation of the camouflage material solutions is given in the Supporting Information.

### DPV Method Validation against the GC–MS and Spectrophotometric Method Using Contaminated Clay Soil Samples

A volume of 1.25 mL of 1000 mg L^–1^ TATP solution was mixed with 1.0 g of clay soil to prepare the contaminated clay soil sample. Two portions of 10 mL followed by 5 mL of acetone were added to the clay soil sample and kept in an ultrasonic bath for 5 min each time. The contents were taken into a centrifuge tube, centrifuged for 5 min at 5000 rpm, filtered through GF-PET, and transferred to a 25 mL flask with dilution to the mark (the final concentration was 50 mg L^–1^ TATP). The clay soil sample contaminated with HMTD was also prepared as mentioned above, and its final concentration was 50 mg L^–1^. Later, clay soil samples contaminated with PBEs were diluted 100-fold with acetone, and each was determined by the proposed DPV method.

For the validation of the proposed DPV method of TATP determination against the GC–MS method,^28^ the contaminated sample was diluted 10-fold with acetone. The GC system was equipped with a Thermo 5MS column (30 m × 320 μm × 0.25 μm), and a volume of 1.0 μL was injected. The injector temperature was 110 °C. The starting temperature of the oven was set to 50 °C, and it was held for 3 min and increased at a rate of 8 °C min^–1^ to a final temperature of 100 °C, which was held for 6 min. The GC–MS interface temperature was 150 °C, the MS source temperature was 200 °C, and the scan *m*/*z* range was 30–300.

A literature spectrophotometric method was used to validate the method for HMTD.^9^ The prepared HMTD-contaminated soil sample was used directly. With this method, HMTD was converted to H_2_O_2_*via* acidic hydrolysis, and the released H_2_O_2_ was determined using the CUPRAC method, for which the absorbance values were recorded at 454 nm. The DPV method was statistically compared against the GC–MS method for TATP and the spectrophotometric method for HMTD using the t- and F- tests.

## Results and Discussion

### Fabrication of a TATP-Memory-PCz Film on the GCE

**Figure 1:**
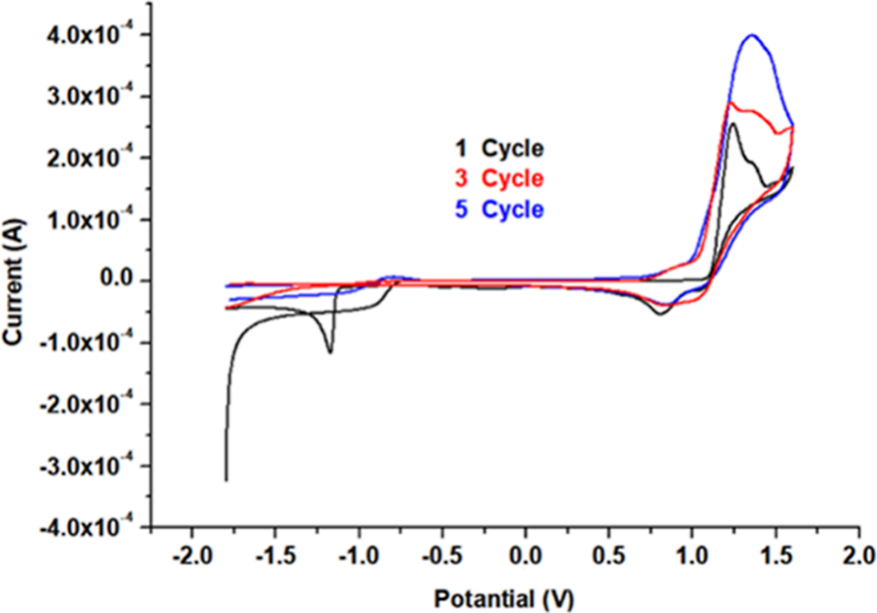
Cyclic voltammograms for the polymerization of 1.0 × 10^–2^ mol L^–1^ Cz containing 100 mg L^–1^ TATP in solution.

The preparation of the TATP-memory-GC/PCz working electrode was carried out in two steps, as described in the Experimental Section. The cyclic voltammograms of the TATP-memory-GC/PCz electrode can be seen in Figure 1, where an oxidation peak at 1.25 V, a reduction peak at 0.78 V for Cz, and a reduction peak at −1.08 V for TATP were obtained. These peaks belong to the cation radical oxidation of the monomers. As the number of cycles increased, the amount of monomer and TATP in the solution medium decreased, while the amount of polymer and TATP accumulated on the electrode surface increased. A similar procedure was used to fabricate the HMTD-memory-GC/PCz electrode, where an oxidation peak at 1.30 V, a reduction peak at 0.80 V for Cz, and a reduction peak at −1.15 V for HMTD were obtained (Figures S4 and S5).

**Figure 2:**
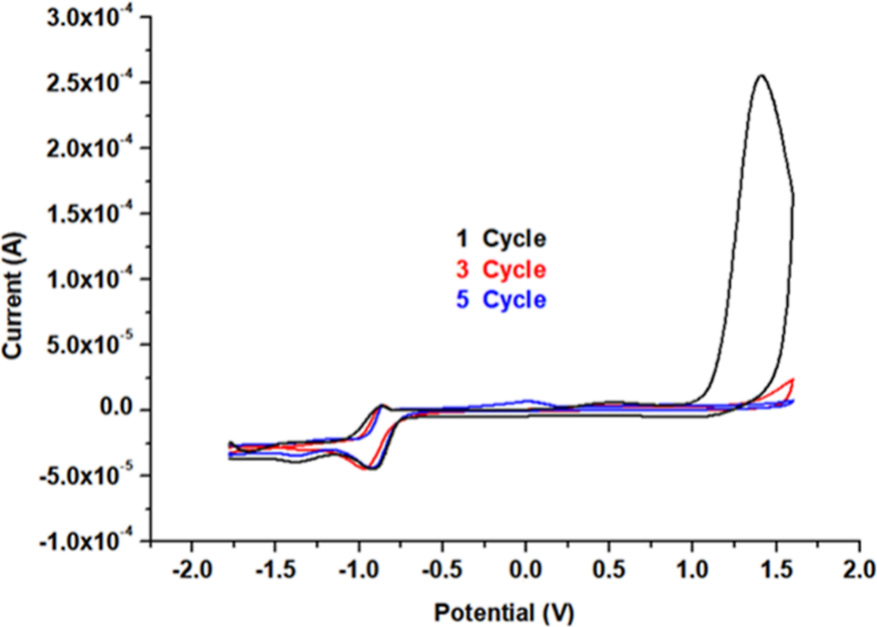
Stability control voltammograms of the TATP-memory-GC/PCz-modified working electrode.

In the second step of the modification, the polymer-coated electrode was stabilized with the CV method as described in the Experimental Section. The current intensities recorded at the end of the first and fifth cycles did not differ considerably. In addition, although the number of cycles varied, the surface-bound TATP amount was found to stabilize on the electrode surface (as observed from the TATP reduction peak current) (Figure 2).

### AuNPs—Modification on the TATP-Memory-GC/PCz-Modified Electrode

The purpose of modifying the surface of the TATP-memory-GC/PCz-modified working electrode with AuNPs was to protect the polymer layer on the electrode surface and to increase its conductivity to allow more sensitive determination of the target energetic materials.

**Figure 3:**
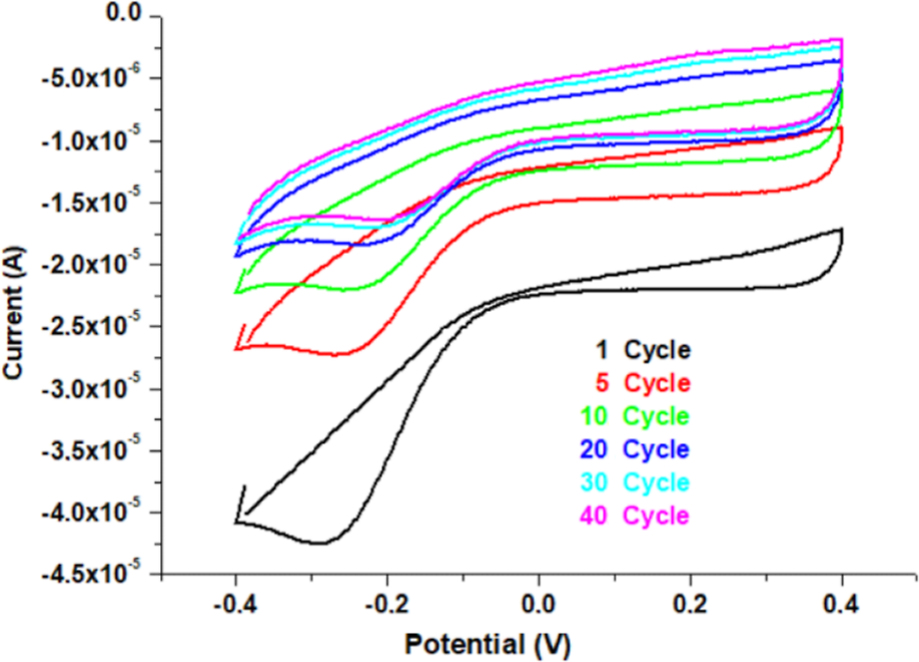
Cyclic voltammograms of AuNPs on TATP-memory-GC/PCz.

As can be seen in Figure 3, a peak of Au^3+^ reduction to AuNPs was monitored around −0.28 V in the first cycle of AuNPs deposition on the TATP-memory-GC/PCz-modified electrode surface. As the number of cycles increased, the current due to the remaining Au^3+^ ions decreased because trivalent gold was reduced to AuNPs and deposited on the electrode surface. After a certain number of cycles (40), the current intensity due to Au^3+^ ions did not change compared to those in the previous cycles, demonstrating that the maximal amount of AuNPs that can accumulate on the electrode surface was reached. The prepared TATP-memory-GC/PCz/AuNPs electrode was used repeatedly throughout the day without any need for cleaning. AuNPs have extra features such as small particle size, high surface area, good electrical properties, strong adsorption capability, and mechanical/chemical stability. Moreover, AuNPs can be used to increase the current response, determination sensitivity, and electronic transmission.^34−36^ The modification of the HMTD-memory-GC/PCz electrode with AuNPs was carried out with a similar procedure, whose details are given in the Supporting Information (Figure S6).

### Electrochemical Determination of TATP and HMTD

**Scheme 1:**
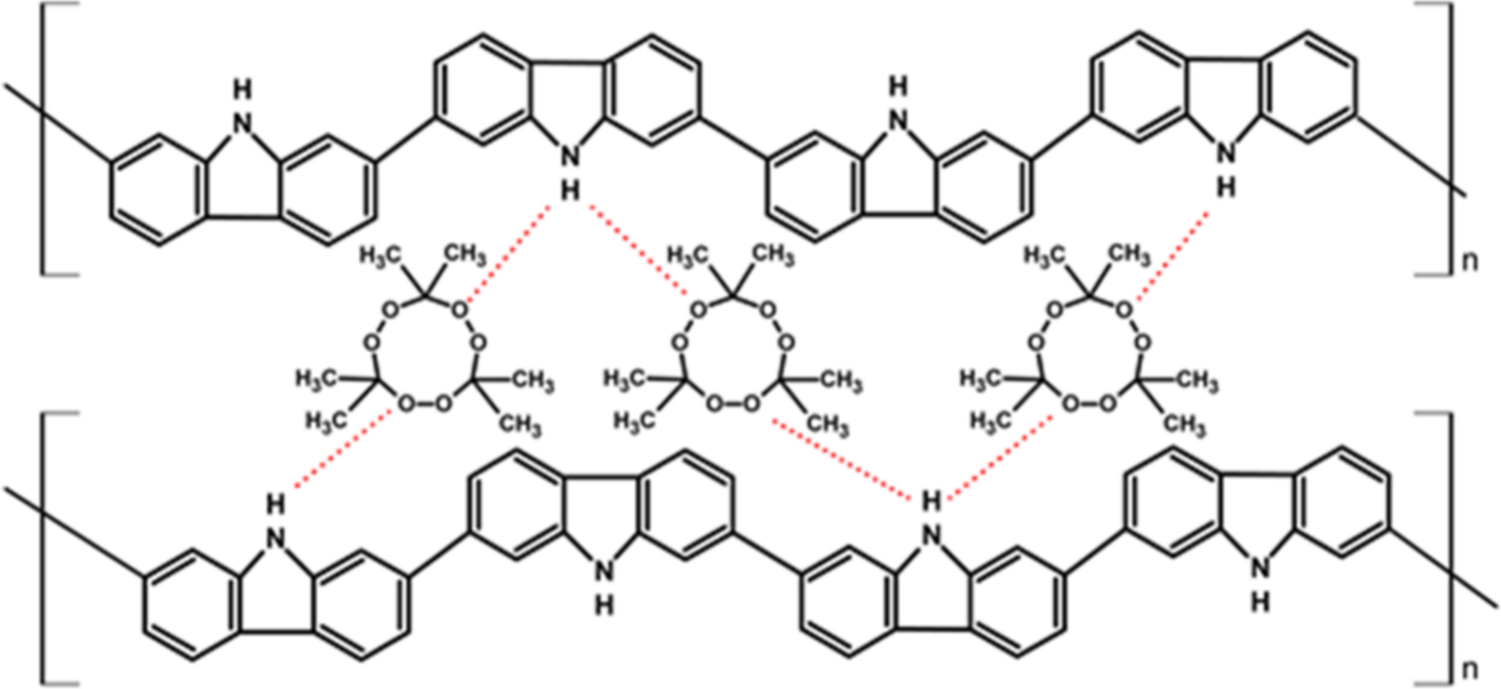
Mechanism of Hydrogen Bond Formation between TATP and PCz

Electrochemical analysis of TATP and HMTD is generally carried out indirectly (converting their bound peroxides into H_2_O_2_ under appropriate conditions), and there are quite a few studies based on their direct determination in the literature. In this work, the detection mechanism of the developed MIP sensor electrode is based on the formation of hydrogen bonds between the oxygen atoms in the peroxide (−C–O–O–C−) bond of TATP and HMTD molecules and the hydrogen atom of the N–H group in the carbazole units of the polymer (Scheme 1). This hydrogen bonding contributes to MIP selectivity because it is assumed to lead to branching and cross-linking in the electropolymerized substrate to generate a three-dimensional matrix with niches containing the template molecule at the right orientation, where the imprinting process is thought to create a microenvironment for the recognition of the analyte depending on shape selection and positioning of the functional groups.^37^

**Figure 4:**
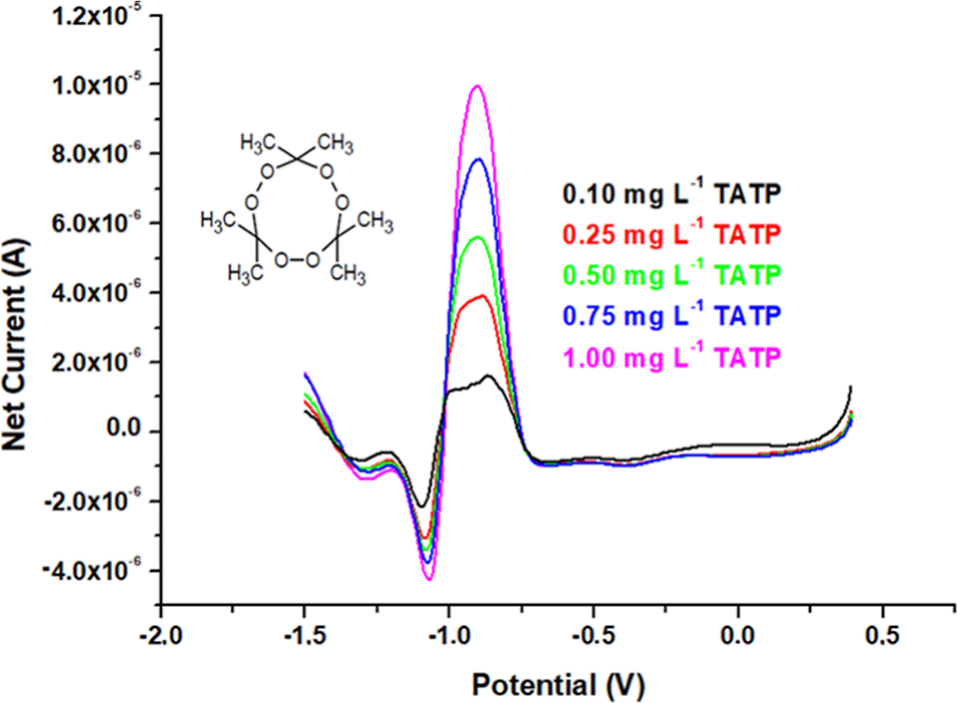
Differential pulse voltammograms and structure of TATP as an inset figure recorded with the TATP-memory-GC/PCz/AuNPs electrode.

**Figure 5:**
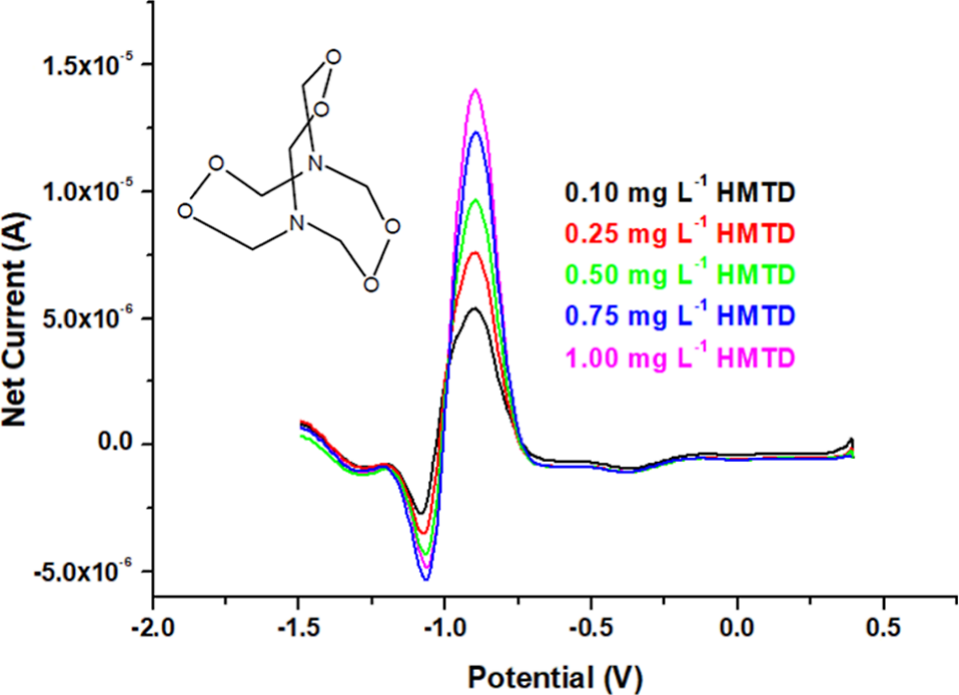
Differential pulse voltammograms and structure of HMTD as an inset figure recorded with the HMTD-memory-GC/PCz/AuNPs electrode.

Direct electrochemical determination of TATP (using the TATP-memory-GC/PCz/AuNPs electrode) and HMTD (using HMTD-memory-GC/PCz/AuNPs) was performed within the concentration range of 0.1–1.0 mg L^–1^ by the proposed DPV method in a potential range from 0.4 to −1.6 V in the presence of 0.025 mol L^–1^ TBABF_4_ as the supporting electrolyte. The DPV voltammograms and structures of the PBEs could be seen in Figures 4 and 5, and the reduction peak potential of TATP and HMTD appeared at −0.95 and −0.93 V, respectively. The voltammetric measurements of PBEs were made by using the calibration lines (*A* = *mC* + *n*) with the analytical performance figures [i.e., limit of detection (LOD) = 3σ_bl_/*m* and limit of quantification (LOQ) = 10σ_bl_/*m*, where σ_bl_ denotes the standard deviation of a blank and *m* the slope of the calibration curve]. The calibration lines were formed using net current intensities (Δ*I*, found by subtracting the background signal from the obtained result at the end of the measurement) *versus* concentrations. The results without baseline correction are given in Figures S7 and S8. The three processes leading to the voltammetric background signal build-up are charging of the electrical double layer, faradaic reactions of impurities (e.g., of the electrolyte ions adsorbed in the pores of the electrode surface), and possible oxidation/reduction of the electrode surface.^38^ Since an appreciable increase in background current is usually associated with a higher value of capacitive (non-Faradaic) current^39^ and acetone may affect the characteristics of a double-layer capacitor by giving rise to a relatively low capacitance but high pore resistance of the electrode,^40^ acetone gave the lowest and stable current response among some common solvents and was consequently selected as the optimal solvent for the square-wave voltammetric analysis of cosmetic products using a GC electrode.^41^ In our case, the background peak in routine analyses was assumed to emerge from the baseline solution medium and did not significantly vary to affect measurements.

The calibration equation of TATP (at −0.95 V) and HMTD (at −0.93 V) gave a linear dependence of net reduction current (Δ*I*) *versus* concentration
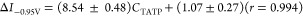

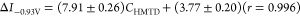
where Δ*I* is the net reduction peak current intensity (μA) and *C*_TATP_ and *C*_HMTD_ are the TATP and HMTD concn. (mg L^–1^). The LOD and LOQ for TATP and HMTD were 15 and 50 μg L^–1^, respectively.

The coefficients of variation of intra- and inter-assay measurements for TATP were 4.7 and 8.6% and for HMTD were 5.4 and 9.3%, respectively. Moreover, the prepared MIP-based sensor electrodes could be used without an additional cleaning process all day, and the service life was 2 days with 88.2% recovery. Additionally, the detection performances of our work were compared to those of articles recently published in Table S1.

### Analysis of Synthetic and Real Energetic Material Mixtures

It is very important to perform a fast and sensitive quantitative analysis of peroxide-type energetic materials in post-blast residues, possibly in the presence of traces of nitro-aromatic, nitramine, and nitrate ester-type explosives. Tests for the analysis of traces of PBEs at a criminologic site should be sufficiently sensitive to detect the analytes at tens of μg L^–1^ level because of the rapid sublimation of TATP from latex, floor, and soil, which was demonstrated in a chemiluminescent assay having 40 μg L^–1^ (for both TATP and HMTD) where TATP remains could only be detected on metal surfaces (but not on other surfaces) after a controlled PBE explosion.^42^ In our work, 0.5 mg L^–1^ TATP and HMTD was analyzed in the presence of a 10-fold concentration of TNT, DNT, tetryl, PA, RDX, HMX, PETN, Comp B, and Octol with the PBE-memory-GC/PCz/AuNPs electrodes using the DPV method. The recoveries of TATP and HMTD were found in the range of 96.37–105.60 and 95.36–105.36%, respectively. All results are given in Figure S9.

### Investigation of the Interference of Electroactive Camouflage Materials

An amount of 0.5 mg L^–1^ TATP and HMTD was analyzed separately using the prepared sensor electrodes in the presence of camouflage materials such as paracetamol, caffeine, d-glucose, acetylsalicylic acid, detergent (containing perborate and percarbonate), and aspartame which did not interfere with the determination of TATP and HMTD at 200-fold (12-fold for caffeine). Besides these electroactive materials, the analyses of TATP and HMTD were also made in the presence of 3-fold concentrations of H_2_O_2_, which proved not to interfere with the determinations. The recoveries of TATP and HMTD were between 98.29–106.71 and 96.22–104.80%, respectively (Figure S10).

### Analytical Results for Contaminated Clay Soil Samples

The TATP-contaminated soil sample was used for the validation of the proposed DPV method against the reference GC–MS method,^28^ and TATP working solutions at 1–10 mg L^–1^ concentrations in acetone were analyzed with GC–MS (the mean value of three repetitive injections was used for each calculation). The calibration equation between the peak area (*A*) and concentration (*C*) was
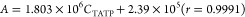


Due to the differences between the sensitivity of methods, the TATP-contaminated soil extract was diluted 10-fold before GC–MS measurements (*N* = 5 repetitive determinations) and 100-fold before the DPV measurements. The mean value of the results obtained using the reference method was 49.4 mg L^–1^ with 98.8% recovery and that obtained using the DPV method was 50.3 mg L^–1^ with 100.6% recovery for TATP.

The HMTD-contaminated soil sample was used for the validation of the proposed DPV method against the reference spectrophotometric method,^9^ and HMTD working solutions at 10–100 mg L^–1^ concentrations were analyzed. The calibration equation was
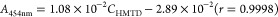


The HMTD-contaminated soil extract was analyzed directly with the spectrophotometric measurements (*N* = 5 repetitive determinations) and diluted 100-fold before the proposed DPV measurements. The mean value of the results obtained using the reference method was 45.0 mg L^–1^ with 90.2% recovery and that obtained using the DPV method was 45.9 mg L^–1^ with 91.9% recovery for HMTD. Statistical *t*- and *F*-tests were used to compare the proposed DPV method and reference methods, and no significant difference was observed between the precision and accuracy of the obtained results at the 95% confidence level (Table 1).

**Table 1:** Statistical Comparison of the Developed Method with Reference Methods for TATP and HMTD Determinations in Contaminated Clay Soil Samples

sample (analyte)	method	mean conc., (mg L^–1^)	Std. dev. (σ)	*S*^a^^,^^b^	*t*^a^^,^^b^	*t*_table_^b^	*F*^b^	*F*_table_^b^
TATP	DPV Method	50.32	0.637					
	GC–MS	49.40	0.706	0.672	2.174	2.306	1.23	6.39
HMTD	DPV Method	45.97	0.938					
	Spectrophotometric Method	45.01	0.460	0.739	1.671	2.306	4.16	6.39

a *S*^2^ = ((*n*_1_ – 1)*s*_1_^2^ + (*n*_2_ – 1)*s*_2_^2^)/(*n*_1_ + *n*_2_ – 2) and *t* = (*a̅*_1_ – *a̅*_2_)/(*S*(1/*n*_1_ + 1/*n*_2_)^1/2^), where *S* is the pooled standard deviation, *s*_1_ and *s*_2_ are the standard deviations of the two populations with sample sizes of *n*_1_ and *n*_2_ and sample means of *a̅*_1_ and *a̅*_2_, respectively (*t* has (*n*_1_ + *n*_2_ – 2) degrees of freedom); here, *n*_1_ = *n*_2_ = 5.

b Statistical comparison made on the paired data produced with developed and reference methods; the results are given only on the row of the reference method.

## Conclusions

Although PBEs are not used for military and industrial purposes due to their unstable nature, they are very dangerous for public security because the starting materials used in their synthesis (such as acetone and hydrogen peroxide) are easily accessible. Although rapid/precise determination of these explosives in the field is essential for public health and security, spectroscopic methods in the literature cannot be directly applied to the assay of TATP and HMTD because they are based on the indirect determination of these explosives by the analysis of H_2_O_2_ released through PBE decomposition. Most of the PBE determinations made by voltammetric techniques are based on indirect determination similar to spectrophotometric methods. The direct electroanalytical method of this work enabled the determination of intact PBEs without degradation, and it did not respond to hydrogen peroxide.

To summarize, a novel molecularly imprinted PBE-memory electrochemical sensor was developed for the direct, ultrasensitive, and selective determination of TATP and HMTD by the proposed DPV method. The method is important as being the first to directly detect TATP and HMTD with a molecular memory electrode with sufficient selectivity and sensitivity relevant for environmental samples. Molecular imprinted techniques are used to prepare materials with cavities that can recognize a particular molecule in terms of shape, size, and functional group. The greatest advantage of MIPs is their high affinity for the target molecule and high selectivity for the analyte over other similar substances. The developed electrochemical sensor electrodes were prepared in two steps. First, the GC electrode surface was coated with a Cz monomer and a template molecule (TATP or HMTD) *via* electrochemical polymerization using the CV method. Then, the PBE-memory-PCz electrode surface was decorated with AuNPs to increase the electrical conductivity and to protect the electrode surface using the CV method. The developed TATP-memory-GC/PCz/AuNPs electrode and the HMTD-memory-GC/PCz/AuNPs electrode were characterized using CV scans, SEM, and electrochemical impedance measurements. The detection selectivity of the developed MIP electrochemical sensor electrode is presumed to arise from the formation of hydrogen bonds between the oxygen atoms in the peroxide (−C–O–O–C−) bond of TATP or HMTD and the hydrogen atom of the N–H group in the Cz units of the polymer.

At optimized experimental parameters, the electrochemical sensor electrodes showed excellent performance for direct TATP and HMTD determination within a concentration range of 0.1–1.0 mg L^–1^ with an LOD of 15 μg L^–1^*via* measuring the cathodic peak currents. The service life of the sensor electrodes was 2 days with a reasonable recovery. Inter- and intra-assay measurements for PBEs lay between 5 and 9%, respectively. Furthermore, TATP and HMTD could both be determined in the presence of different types of explosive materials such as TNT, DNT, tetryl, PA, RDX, HMX, PETN, Comp B, and Octol with the proposed DPV method. In addition, the interference effect of some passenger belongings (analgesic drug, sweetener, sugar, and detergent) that can be used for camouflage purposes was investigated, and the recoveries of TATP and HMTD in the presence of interferents were between 98 and 106%. The proposed DPV method was statistically validated against a reference GC–MS method for TATP and a validated spectrophotometric method for HMTD. Finally, compared to the literature, the developed PBE-memory-GC/PCz/AuNPs sensor electrode and the proposed DPV method were ultrasensitive, selective, simple, less expensive, and precise and allowed on-site/in-field analysis.

## Supporting Information Available

Characterization of the TATP-memory-GC/PCz/AuNPs working electrode, preparation of the camouflage material solutions, fabrication of the HMTD-memory-GC/PCz electrode on the GCE, Au nanoparticle modification on the HMTD-memory-GC/PCz modified electrode, electrochemical determination results of TATP and HMTD without baseline correction, comparison of detection performances of our work with those of other published articles, results of synthetic and real energetic material mixture analysis, and results of interference analysis of electroactive camouflage materials (PDF)

## References

[ref1] Bellamy A. Triacetone Triperoxide: Its Chemical Destruction. J. Forensic Sci. 1999, 44, 14517J 10.1520/JFS14517J.

[ref2] Schulte-Ladbeck R.; Edelmann A.; Quintás G.; Lendl B.; Karst U. Determination of Peroxide-Based Explosives Using Liquid Chromatography with on-Line Infrared Detection. Anal. Chem. 2006, 78, 8150–8155. 10.1021/ac0609834. 17134152

[ref3] Munoz R. A. A.; Lu D.; Cagan A.; Wang J. “One-Step Simplified Electrochemical Sensing of TATP Based on Its Acid Treatment. Analyst 2007, 132, 560–565. 10.1039/b701356f. 17525813

[ref4] Cotte-Rodríguez I.; Chen H.; Cooks R. G. Rapid Trace Detection of Triacetone Triperoxide (TATP) by Complexation Reactions during Desorption Electrospray Ionization. Chem. Commun. 2006, 9, 953–955. 10.1039/b515122h. 16491173

[ref5] Parajuli S.; Miao W. Sensitive Determination of Triacetone Triperoxide Explosives Using Electrogenerated Chemiluminescence. Anal. Chem. 2013, 85, 8008–8015. 10.1021/ac401962b. 23885721

[ref6] Engel Y.; Elnathan R.; Pevzner A.; Davidi G.; Flaxer E.; Patolsky F. Supersensitive Detection of Explosives by Silicon Nanowire Arrays. Angew. Chem., Int. Ed. 2010, 49, 6830–6835. 10.1002/ANIE.201000847. 20715224

[ref7] Lichtenstein A.; Havivi E.; Shacham R.; Hahamy E.; Leibovich R.; Pevzner A.; Krivitsky V.; Davivi G.; Presman I.; Elnathan R.; Engel Y.; Flaxer E.; Patolsky F. Supersensitive Fingerprinting of Explosives by Chemically Modified Nanosensors Arrays. Nat. Commun. 2014, 5, 1–12. 10.1038/ncomms5195. 24960270

[ref8] Dubnikova F.; Kosloff R.; Zeiri Y.; Karpas Z. Novel Approach to the Detection of Triacetone Triperoxide (TATP): Its Structure and Its Complexes with Ions. J. Phys. Chem. A 2002, 106, 4951–4956. 10.1021/jp014189s.

[ref9] Eren Ş.; Üzer A.; Can Z.; Kapudan T.; Erçağ E.; Apak R. Determination of Peroxide -Based Explosives with Copper(Ii) – Neocuproine Assay Combined with a Molecular Spectroscopic Sensor. Analyst 2010, 135, 2085–2091. 10.1039/B925653A. 20532268

[ref10] Can Z.; Üzer A.; Türkekul K.; Erçağ E.; Apak R. Determination of Triacetone Triperoxide with a N,N-Dimethyl-p-Phenylenediamine Sensor on Nafion Using Fe3O4 Magnetic Nanoparticles. Anal. Chem. 2015, 87, 9589–9594. 10.1021/acs.analchem.5b01775. 26356315

[ref11] Malashikhin S.; Finney N. S. Fluorescent Signaling Based on Sulfoxide Profluorophores: Application to the Visual Detection of the Explosive TATP. J. Am. Chem. Soc. 2008, 130, 12846–12847. 10.1021/JA802989V/SUPPL_FILE/JA802989V_SI_001.PDF. 18774810

[ref12] Schulte-Ladbeck R.; Karst U. Determination of Triacetonetriperoxide in Ambient Air. Anal. Chim. Acta 2003, 482, 183–188. 10.1016/S0003-2670(03)00212-5.

[ref13] Widmer L.; Watson S.; Schlatter K.; Crowson A. Development of an LC/MS Method for the Trace Analysis of Triacetone Triperoxide (TATP). Analyst 2002, 127, 1627–1632. 10.1039/B208350G. 12537371

[ref14] Sigman M. E.; Clark C. D.; Fidler R.; Geiger C. L.; Clausen C. A. Analysis of Triacetone Triperoxide by Gas Chromatography/Mass Spectrometry and Gas Chromatography/Tandem Mass Spectrometry by Electron and Chemical Ionization. Rapid Commun. Mass Spectrom. 2006, 20, 2851–2857. 10.1002/RCM.2678. 16941533

[ref15] McKay G. J. Forensic Characteristics of Organic Peroxide Explosives (TATP, DADP and HMTD). Kayaku Gakkaishi 2002, 63, 323–329.

[ref16] Guo C. X.; Lei Y.; Li C. M. Porphyrin Functionalized Graphene for Sensitive Electrochemical Detection of Ultratrace Explosives. Electroanalysis 2011, 23, 885–893. 10.1002/ELAN.201000522.

[ref17] Krivitsky V.; Filanovsky B.; Naddaka V.; Patolsky F. Direct and Selective Electrochemical Vapor Trace Detection of Organic Peroxide Explosives via Surface Decoration. Anal. Chem. 2019, 91, 5323–5330. 10.1021/acs.analchem.9b00257. 30892020

[ref18] Mamo S. K.; Gonzalez-Rodriguez J. Development of a Molecularly Imprinted Polymer-Based Sensor for the Electrochemical Determination of Triacetone Triperoxide (TATP). Sensors 2014, 14, 23269–23282. 10.3390/s141223269. 25490589 PMC4299062

[ref19] Alizadeh T. Preparation of Magnetic TNT-Imprinted Polymer Nanoparticles and Their Accumulation onto Magnetic Carbon Paste Electrode for TNT Determination. Biosens. Bioelectron. 2014, 61, 532–540. 10.1016/j.bios.2014.05.041. 24951924

[ref20] Caygill J. S.; Davis F.; Higson S. P. J. Current Trends in Explosive Detection Techniques. Talanta 2012, 88, 14–29. 10.1016/J.TALANTA.2011.11.043. 22265465

[ref21] Leibl N.; Duma L.; Gonzato C.; Haupt K. Polydopamine-Based Molecularly Imprinted Thin Films for Electro-Chemical Sensing of Nitro-Explosives in Aqueous Solutions. Bioelectrochemistry 2020, 135, 107541 10.1016/j.bioelechem.2020.107541. 32388439

[ref22] Guo Z. Z.; Florea A.; Cristea C.; Bessueille F.; Vocanson F.; Goutaland F.; Zhang A. D.; Săndulescu R.; Lagarde F.; Jaffrezic-Renault N. 1,3,5-Trinitrotoluene Detection by a Molecularly Imprinted Polymer Sensor Based on Electropolymerization of a Microporous-Metal-Organic Framework. Sens. Actuators, B 2015, 207, 960–966. 10.1016/j.snb.2014.06.137.

[ref23] Alizadeh T.; Atashi F.; Ganjali M. R. Molecularly Imprinted Polymer Nano-Sphere/Multi-Walled Carbon Nanotube Coated Glassy Carbon Electrode as an Ultra-Sensitive Voltammetric Sensor for Picomolar Level Determination of RDX. Talanta 2019, 194, 415–421. 10.1016/j.talanta.2018.10.040. 30609552

[ref24] Sağlam Ş.; Üzer A.; Erçağ E.; Apak R. Electrochemical Determination of TNT, DNT, RDX, and HMX with Gold Nanoparticles/Poly(Carbazole-Aniline) Film-Modified Glassy Carbon Sensor Electrodes Imprinted for Molecular Recognition of Nitroaromatics and Nitramines. Anal. Chem. 2018, 90, 7364–7370. 10.1021/acs.analchem.8b00715. 29786423

[ref25] Laine D. F.; Roske C. W.; Cheng I. F. Electrochemical Detection of Triacetone Triperoxide Employing the Electrocatalytic Reaction of Iron(II/III)-Ethylenediaminetetraacetate and Hydrogen Peroxide. Anal. Chim. Acta 2008, 608, 56–60. 10.1016/j.aca.2007.12.003. 18206994

[ref26] Lu D.; Cagan A.; Munoz R. A. A.; Tangkuaram T.; Wang J. Highly Sensitive Electrochemical Detection of Trace Liquid Peroxide Explosives at a Prussian-Blue “artificial-Peroxidase” Modified Electrode. Analyst 2006, 131, 1279–1281. 10.1039/b613092e. 17124534

[ref27] Arman A.; Sağlam Ş.; Üzer A.; Apak R. Direct Electrochemical Determination of Peroxide-Type Explosives Using Well-Dispersed Multi-Walled Carbon Nanotubes/Polyethyleneimine-Modified Glassy Carbon Electrodes. Anal. Chem. 2021, 93, 11451–11460. 10.1021/acs.analchem.1c01397. 34425678

[ref28] Räsänen R. M.; Nousiainen M.; Peräkorpi K.; Sillanpää M.; Polari L.; Anttalainen O.; Utriainen M. Determination of Gas Phase Triacetone Triperoxide with Aspiration Ion Mobility Spectrometry and Gas Chromatography-Mass Spectrometry. Anal. Chim. Acta 2008, 623, 59–65. 10.1016/j.aca.2008.05.076. 18611458

[ref29] Schulte-Ladbeck R.; Kolla P.; Karst U. Trace Analysis of Peroxide-Based Explosives. Anal. Chem. 2003, 75, 731–735. 10.1021/AC020392N. 12622359

[ref30] Dubnikova F.; Kosloff R.; Almog J.; Zeiri Y.; Boese R.; Itzhaky H.; Alt A.; Keinan E. Decomposition of Triacetone Triperoxide Is an Entropic Explosion. J. Am. Chem. Soc. 2005, 127, 1146–1159. 10.1021/ja0464903. 15669854

[ref31] Schaefer W. P.; Fourkas J. T.; Tiemann B. G. Structure of Hexamethylene Triperoxide Diamine. J. Am. Chem. Soc. 1985, 107, 2461–2463. 10.1021/ja00294a043.

[ref32] Arman A.; Üzer A.; Sağlam Ş.; Erçağ E.; Apak R. Indirect Electrochemical Determination of Antioxidant Capacity with Hexacyanoferrate(III) Reduction Using a Gold Nanoparticle-Coated o-Phenylenediamine-Aniline Copolymer Electrode. Anal. Lett. 2019, 52, 1282–1297. 10.1080/00032719.2018.1536137.

[ref33] Sağlam Ş.; Üzer A.; Tekdemir Y.; Erçağ E.; Apak R. Electrochemical Sensor for Nitroaromatic Type Energetic Materials Using Gold Nanoparticles/Poly(o-Phenylenediamine-Aniline) Film Modified Glassy Carbon Electrode. Talanta 2015, 139, 181–188. 10.1016/j.talanta.2015.02.059. 25882425

[ref34] Lian W.; Liu S.; Yu J.; Xing X.; Li J.; Cui M.; Huang J. Electrochemical Sensor Based on Gold Nanoparticles Fabricated Molecularly Imprinted Polymer Film at Chitosan-Platinum Nanoparticles/Graphene-Gold Nanoparticles Double Nanocomposites Modified Electrode for Detection of Erythromycin. Biosens. Bioelectron. 2012, 38, 163–169. 10.1016/j.bios.2012.05.017. 22683249

[ref35] Matharu Z.; Daggumati P.; Wang L.; Dorofeeva T. S.; Li Z.; Seker E. Nanoporous-Gold-Based Electrode Morphology Libraries for Investigating Structure-Property Relationships in Nucleic Acid Based Electrochemical Biosensors. ACS Appl. Mater. Interfaces 2017, 9, 12959–12966. 10.1021/ACSAMI.6B15212/SUPPL_FILE/AM6B15212_SI_001.PDF. 28094510

[ref36] Xue C.; Han Q.; Wang Y.; Wu J.; Wen T.; Wang R.; Hong J.; Zhou X.; Jiang H. Amperometric Detection of Dopamine in Human Serumby Electrochemical Sensor Based on Gold Nanoparticles Doped Molecularly Imprinted Polymers. Biosens. Bioelectron. 2013, 49, 199–203. 10.1016/J.BIOS.2013.04.022. 23747995

[ref37] Özcan L.; Şahin Y. Determination of Paracetamol Based on Electropolymerized-Molecularly Imprinted Polypyrrole Modified Pencil Graphite Electrode. Sens. Actuators, B 2007, 127, 362–369. 10.1016/j.snb.2007.04.034.

[ref38] Sokol W. F.; Evans D. H. Suppression of Background Current in Differential Pulse Voltammetry with Solid Electrodes. Anal. Chem. 1981, 53, 578–580. 10.1021/ac00227a004.

[ref39] Porada R.; Fendrych K.; Baś B. The Mn-zeolite/Graphite Modified Glassy Carbon Electrode: Fabrication, Characterization and Analytical Applications. Electroanalysis 2020, 32, 1208–1219. 10.1002/elan.201900744.

[ref40] Arulepp M.; Permann L.; Leis J.; Perkson A.; Rumma K.; Jänes A.; Lust E. Influence of the Solvent Properties on the Characteristics of a Double Layer Capacitor. J. Power Sources 2004, 133, 320–328. 10.1016/j.jpowsour.2004.03.026.

[ref41] Jashari G.; Musliu A.; Sýs M.; Arbneshi T.; Mikysek T.; Švancara I.; Metelka R. Simultaneous Determination of Lipophilic Vitamin Esters Using Square-wave Voltammetry at the Glassy Carbon Electrode. Electroanalysis 2021, 33, 537–542. 10.1002/elan.202060302.

[ref42] Girotti S.; Ferri E.; Maiolini E.; Bolelli L.; D’Elia M.; Coppe D.; Romolo F. S. A Quantitative Chemiluminescent Assay for Analysis of Peroxide-Based Explosives. Anal. Bioanal. Chem. 2011, 400, 313–320. 10.1007/s00216-010-4626-3. 21249343

