# Supplementary material for: Direct Determination of Peroxide Explosives on Polycarbazole/Gold Nanoparticle-Modified Glassy Carbon Sensor Electrodes Imprinted for Molecular Recognition of TATP and HMTD
Source: Anal Chem. 2022 Dec 6;94(50):17662–9. doi: 10.1021/acs.analchem.2c04450 (PMC9773174; doi:10.1021/acs.analchem.2c04450)
Supplement: Supplementary file 1 — ac2c04450_si_001.pdf [file ac2c04450_si_001.pdf]

SUPPORTING INFORMATION

Direct Determination of Peroxide Explosives on Polycarbazole/Gold Nanoparticle−Modified Glassy Carbon Sensor Electrodes Imprinted for Molecular Recognition of TATP and HMTD

Şener Sağlam[1], Ayşem Üzer[1, *], Reşat Apak[1,2, *]

[1]Engineering Faculty, Chemistry Department, Istanbul University−Cerrahpaşa, 34320 Avcilar, Istanbul, Turkey

[2]Turkish Academy of Sciences (TUBA), Bayraktar neighborhood, Vedat Dalokay st. No:112, 06670, Cankaya, Ankara, Turkey

* Corresponding author; E-mail: auzer@iuc.edu.tr

* Co-corresponding author; E-mail: rapak@istanbul.edu.tr

Supporting information contains; *(i) Characterization of TATP-memory-GC/PCz/AuNPs working electrode, (ii) Preparation of camouflage material solutions, (iii) Fabrication of HMTD-memory-GC/PCz electrode on GCE, (iv) Au nanoparticles modification on HMTD-memory-GC/PCz modified electrode, (v) Electrochemical determination results of TATP and HMTD without baseline correction, (vi) Comparison of detection performances of our work with those of other published articles, (vii) Results of synthetic and real energetic material mixtures analysis, (viii) Results of interference analysis of electroactive camouflage materials*.

(i) Characterization of TATP-memory-GC/PCz/AuNPs Working Electrode

The proposed TATP-memory-GC/PCz/AuNPs modified electrode was characterized *via* cyclic voltammetric scans (CV Scans), scanning electron microscopy (SEM), and electrochemical impedance measurements before the determination of PBEs.

The CV scans were performed using the bare GC, TATP-memory-GC/PCz, TATP-memory-GC/PCz/AuNPs electrodes in a 5 mL solution having the composition of 0.10 mol L^{-1} HCl, 0.10 mol L^{-1} KCl, and 5.0 mmol L^{-1} $[Fe(CN)_6]^{3-/4-}$ with a scan speed of 50 mV s^{-1} in the potential range between −0.6 and 0.6 V (Figure S1).

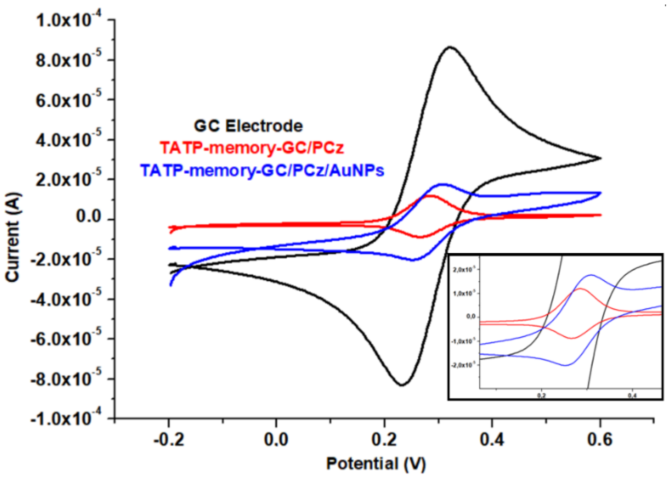

Figure S1. CV scans of bare GC, TATP-memory-GC/PCz, TATP-memory-GC/PCz/AuNPs electrodes in a 5 mL solution having the composition of 0.10 mol L^{-1} HCl, 0.10 mol L^{-1} KCl and 5.0 mmol L^{-1} $[Fe(CN)_6]^{3-/4-}$.

The cathodic and anodic peaks of $[Fe(CN)_6]^{3-/4-}$ redox couple were observed at about 0.23 V and 0.32 V for bare GC electrode, and at about 0.26 V and 0.30 V for TATP-memory-GC/PCz and TATP-memory-GC/PCz/AuNPs electrodes, respectively. The cathodic and anodic peak current values were -82.92 µA and 86.33 µA for the bare GC electrode, -8.75 µA and 12.05 µA for TATP-memory-GC/PCz electrode, and -20.10 µA and 17.70 µA for TATP-memory-GC/PCz/AuNPs electrode, respectively. The reduction-to-oxidation peak potential separation (ΔE_p) was 40 mV for modified electrodes, showing electrode reversibility, and each electrode had electro-activity.

The scanning electron microscopy (SEM) was used for the surface characterization of the TATP-memory-GC/PCz/AuNPs working electrode (SEM; FEI Model Quanta 450 FEG, Hillsboro, OR, USA). As a result of this measurement, it was determined that the sizes of the

gold nanoparticles on the TATP-memory-GC/PCz/AuNPs electrode surface were between 30-250 nm (Figure S2).

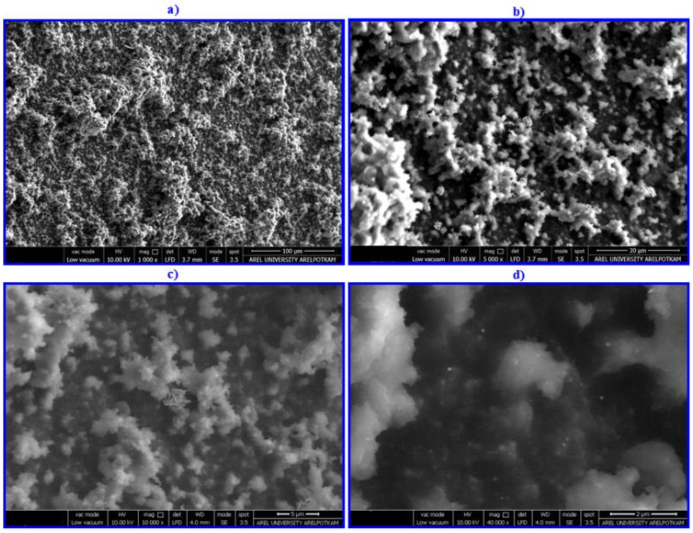

Figure S2. SEM images of TATP-memory-GC/PCz/AuNPs working electrode with (a) 1000-fold, (b) 5000-fold, (c) 10000-fold and (d) 40000-fold zoom.

Conductivity measurements of the bare GC, TATP-memory-GC/PCz, and TATP-memory-GC/PCz/AuNPs working electrodes were performed by the Potentiostat EIS method in the 0.10 mol L^{-1} KCl, 0.10 mol L^{-1} HCl and 5.0 mmol L^{-1} $[Fe(CN)_6]^{3-/4-}$ solution medium; frequency range was 10 mHz – 0.1 MHz, and points/decade 10 mV (Figure S3).

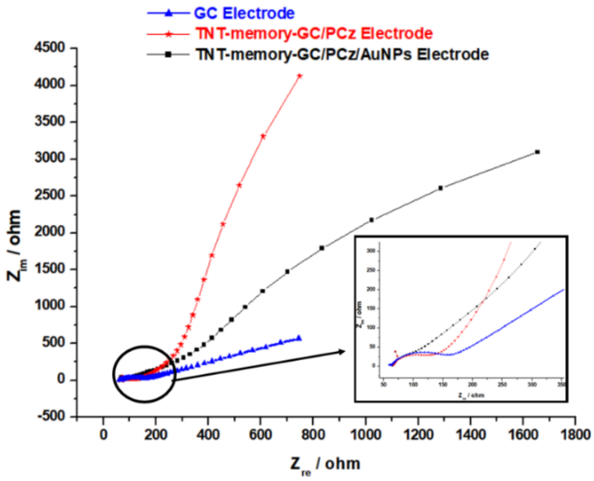

Figure S3. Impedance measurements of bare GC, TATP-memory-GC/PCz, and TATP-memory-GC/PCz/AuNPs working electrodes in the medium of 5.0 mmol L^{-1} $[Fe(CN)_6]^{3-/4-}$ solution containing 0.10 mol L^{-1} KCl and 0.10 mol L^{-1} HCl.

The electrochemical impedance spectroscopy method is used to evaluate the electron transfer properties of the developed electrodes. As a result of these measurements, the R_{ct} (charge transfer resistance, which is the opposite of electron movement) values of the electrodes can be calculated [1]. When Fig. S3 was examined, the largest semi-circle diameter was obtained for the bare GC electrode (it can be seen more detailed in the inset figure), and R_{ct} was 112.90 Ω. The diameter of the semi-circle obtained for the TATP-memory-GC/PCz working electrode was smaller than the bare GC electrode, and its R_{ct} was 77.38 Ω. On the other hand, the R_{ct} value was quite small and no semi-circle was obtained for TATP-memory-GC/PCz/AuNPs electrode, and the lowest R_{ct} value indicated that the developed sensor electrode had the highest electrical conductivity.

(ii) Preparation of Camouflage Material Solutions

Stock solutions of electroactive camouflage materials as potential interferents, which are similar in color and appearance to TATP and HMTD energetic materials, were prepared in acetone solvent at a concentration of 1000 mg L^{-1}. For the preparation of paracetamol and caffeine solution, 33 mg vermidon tablet (one tablet is approximately 700 mg, containing 500 mg paracetamol and 30 mg caffeine) was taken and dissolved in 25 mL acetone in an ultrasonic bath for 15 minutes. Then, this solution was filtered through a Chromafil Xtra PTFE-45/25 filter and diluted to the mark in a 25 mL flask with acetone to yield a solution containing 1000 mg L^{-1} paracetamol and 60 mg L^{-1} caffeine. A mass of 30 mg aspirin (one tablet is approximately 600 mg, containing 500 mg acetylsalicylic acid), 125 mg canderel (one tablet is approximately 90 mg, containing 18 mg aspartame, 25 mg D-Glucose, and 25 mg detergent (containing perborate and percarbonate) were used for the preparation of the corresponding solutions with the same procedure followed for paracetamol and caffeine. The final concentrations in the solution were 1000 mg L^{-1} for each material.

(iii) Fabrication of HMTD-memory-GC/PCz electrode on GCE

The preparation of the HMTD-memory-GC/PCz working electrode was carried out in two steps. In the first step of the modification, 5 mL of a solution containing 1.0×10^{-2} M carbazole (Cz) monomer dissolved in acetonitrile solvent, 100 mg L^{-1} HMTD (used as a template molecule), and 0.1 M TEAP supporting electrolyte were taken into the working cell. The electropolymerization process was carried out with the CV method in the potential range (-1.8 V) - (1.6 V) at a scanning speed of 20 mV s^{-1} and for 5 cycles. The CV of the HMTD-memory-GC/PCz electrode can be seen in Figure S4, where an oxidation peak at 1.30 V, a reduction peak at 0.80 V for Cz, and a reduction peak at -1.15 V for HMTD were obtained.

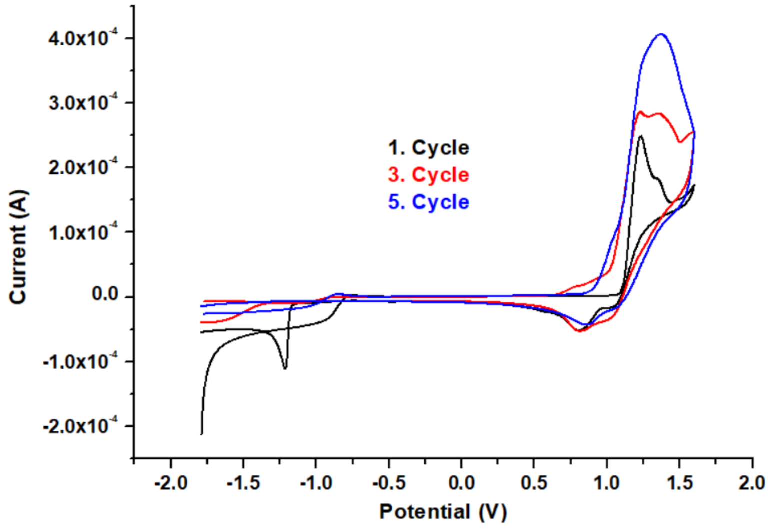

Figure S4. The cyclic voltammograms for polymerization of 1.0×10^{-2} mol L^{-1} Cz containing 100 mg L^{-1} HMTD in solution.

In the second step of the modification, the polymer-coated electrode was stabilized by polymerizing the remaining monomers, dimers, and oligomers on the surface in a supporting electrolyte medium (not containing monomer) within the potential range (-1.8 V) – (1.6 V) at a scanning speed of 20 mVs^{-1} for 5 cycles using the CV method. The currents recorded at the end of the first and fifth cycles did not differ considerably. In addition, although the number of cycles varied, the surface-bound HMTD amount was found to stabilize on the electrode surface (as observed from the HMTD reduction peak current) (Figure S5).

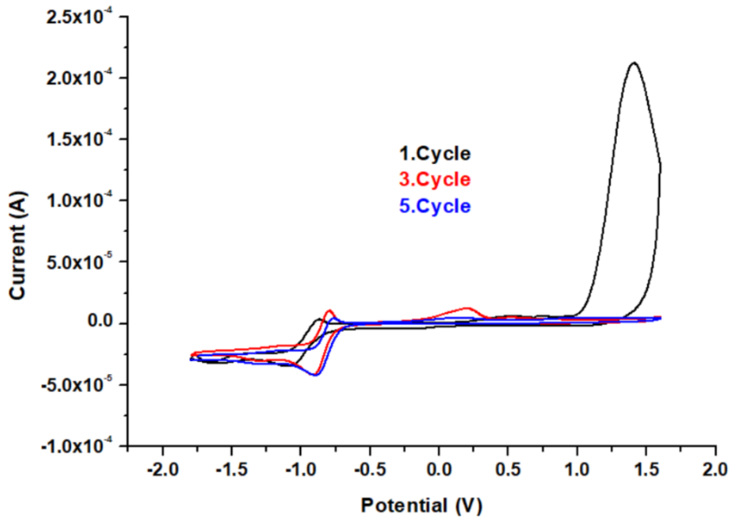

Figure S5. Stability control voltammograms of the HMTD-memory-GC/PCz modified working electrode.

(iv) Au Nanoparticles Modification on HMTD-memory-GC/PCz Modified Electrode

As seen in Figure S6, a peak of Au^{3+} reduction to AuNPs was monitored around -0.20 V in the first cycle of AuNPs deposition on the HMTD-memory-GC/PCz modified electrode surface. As the number of cycles increased, the current due to the remaining Au^{3+} ions decreased as the Au^{3+} were reduced to AuNPs and deposited on the electrode surface. After a certain number of cycles (40), the current value of the Au^{3+} ions did not change compared to the previous cycles. Hence, it was understood that the maximum amount of AuNPs that can accumulate on the electrode surface had been reached. The prepared HMTD-memory-GC/PCz/AuNPs electrode was used repeatedly throughout the day without any need for cleaning.

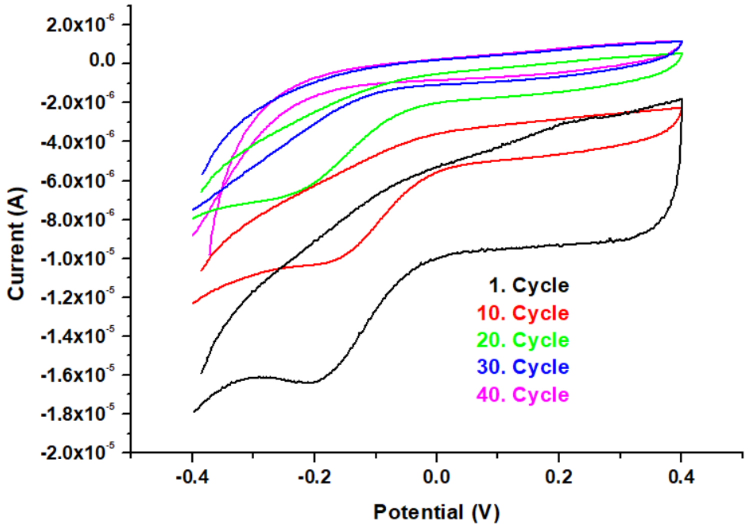

Figure S6. Cyclic voltammograms of AuNPs on HMTD-memory-GC/PCz

(v) Electrochemical Determination Results of TATP and HMTD Without Baseline Correction

Direct electrochemical determination of TATP (using TATP-memory-GC/PCz/AuNPs electrode) and HMTD (using HMTD-memory-GC/PCz/AuNPs) were achieved in the concentration range of 0.1 – 1.0 mg L^{-1} by the proposed DPV method in a potential range from 0.4 V to -1.6 V in the presence of 0.025 mol L^{-1} $TBABF_4$ as supporting electrolyte.

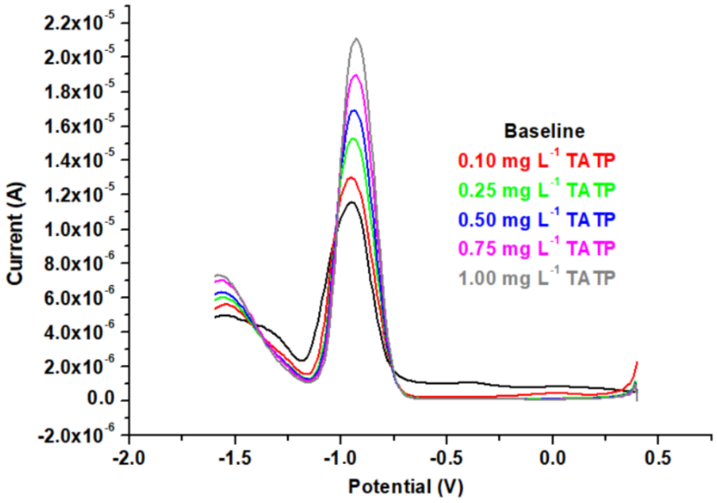

Figure S7. Differential pulse voltammograms of TATP with TATP-memory-GC/PCz/AuNPs electrode without baseline correction.

When Figure S7 was examined, a linear increase was observed in the current values obtained with increasing TATP concentration. In the baseline measurement, a peak was obtained at -0.95 V, and this peak is thought to be caused by acetone containing $TBABF_4$.

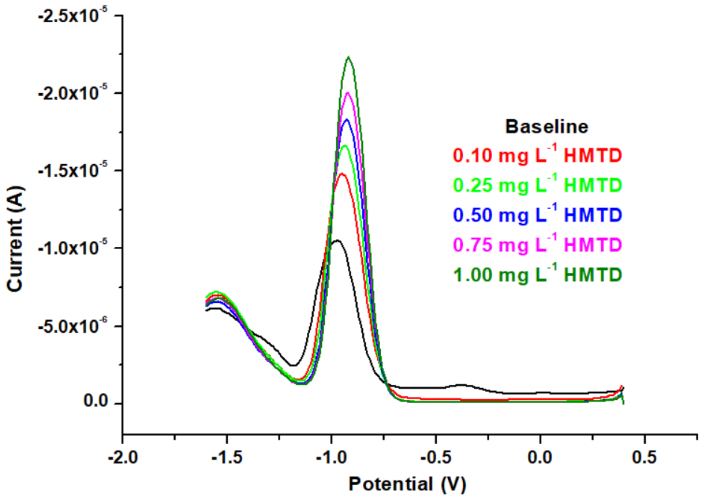

Figure S8. Differential pulse voltammograms of HMTD with HMTD-memory-GC/PCz/AuNPs electrode without baseline correction.

(vi) Comparison of Detection Performances of Our Work With Those of Other Published Articles

Table S1: Comparison of detection performances of our work with those of other articles

**Electrode**	**Method**	**Linear Range (µM)**	**LOD (µM)**	**LOQ (µM)**	**Detection Way**
GCE/ Polypyrrole/TATP-MIP	DPV	0.37 – 199.33	0.12	0.36	Direct [2]
GCE/$Fe^{II/III}$ethylenediaminetetraacetate	CA	0 – 1600	0.89	–	Indirect [3]
GCE/Prussian blue	CA	1– 10	0.049	–	Indirect [4]
AgNPs-decorated µCFE	DPV	135 – 809.9	0.9	–	Direct [5]
Glassy carbon electrode	CA	0 – 100	8.51	–	Indirect [6]
GCE/MWCNTs/PEI	DPV	45 – 900	6.75	22.5	Direct [7]
TATP-memory-GC/PCz/AuNPs electrode	DPV	0.45 – 4.5	0.0675	0.225	Direct (this work)

*GCE: Glassy carbon electrode, CA: Chronoamperometry, AgNPs: Silver nanoparticles, µCFE: Micro carbon fiber electrode, PCz: Polycarbazole, AuNPs: Gold nanoparticles.

(vii) Results of Synthetic and Real Energetic Material Mixtures Analysis

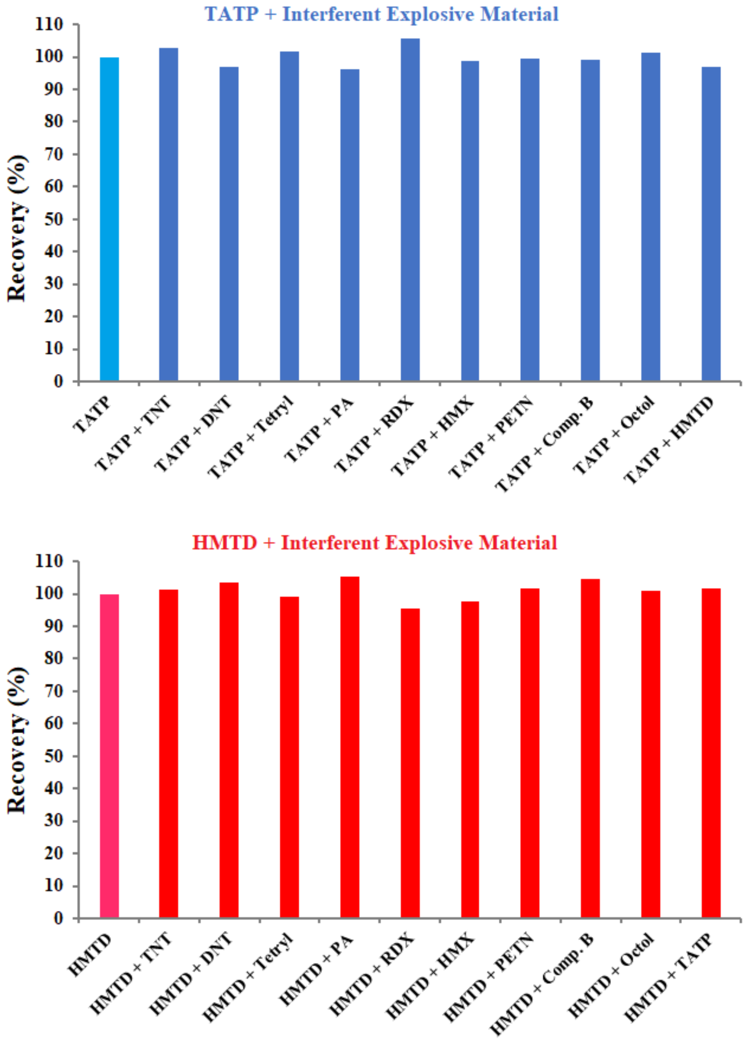

Figure S9. Recovery % of TATP and HMTD in the presence of different energetic materials.

(viii) Results of Interference Analysis of Electroactive Camouflage Materials

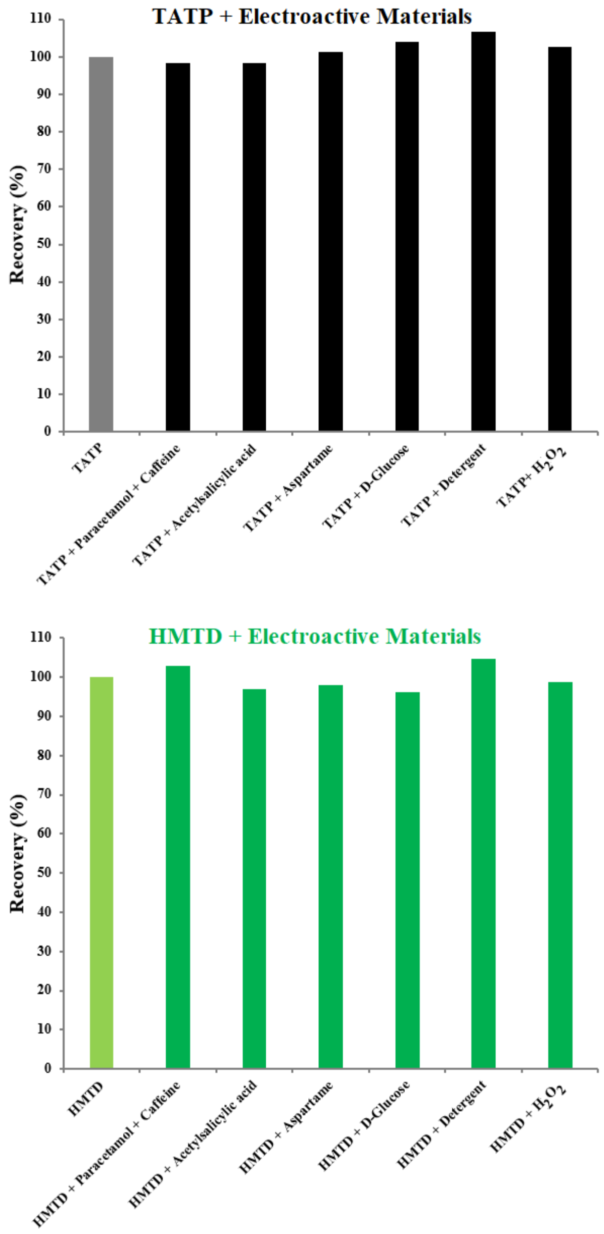

Figure S10. Recovery % of TATP and HMTD in the presence of electroactive materials.

References

(1) Randviir, E. P. ; Banks, C. E. Electrochemical Impedance Spectroscopy: An Overview of Bioanalytical Applications. *Anal. Methods* **2013**, *5* (5), 1098–1115. https://doi.org/10.1039/C3AY26476A.

(2) Mamo, S. K.; Gonzalez-Rodriguez, J. Development of a Molecularly Imprinted Polymer-Based Sensor for the Electrochemical Determination of Triacetone

Triperoxide (TATP). *Sensors* **2014**, *14*, 23269–23282. https://doi.org/10.3390/s141223269.

(3) Laine, D. F.; Roske, C. W.; Cheng, I. F. Electrochemical Detection of Triacetone Triperoxide Employing the Electrocatalytic Reaction of Iron(II/III)-Ethylenediaminetetraacetate and Hydrogen Peroxide. *Anal. Chim. Acta* **2008**, *608* (1), 56–60. https://doi.org/10.1016/j.aca.2007.12.003.

(4) Lu, D.; Cagan, A.; Munoz, R. A. A.; Tangkuaram, T.; Wang, J. Highly Sensitive Electrochemical Detection of Trace Liquid Peroxide Explosives at a Prussian-Blue "artificial-Peroxidase" Modified Electrode. *pubs.rsc.org* **2006**, *131* (12), 1279–1281. https://doi.org/10.1039/b613092e.

(5) Krivitsky, V.; Filanovsky, B.; Naddaka, V.; Patolsky, F. Direct and Selective Electrochemical Vapor Trace Detection of Organic Peroxide Explosives via Surface Decoration. *Anal. Chem.* **2019**, *91* (8), 5323–5330. https://doi.org/10.1021/acs.analchem.9b00257.

(6) Xie, Y.; Cheng, I. F. Selective and Rapid Detection of Triacetone Triperoxide by Double-Step Chronoamperometry. *Microchem. J.* **2010**, *94* (2), 166–170. https://doi.org/10.1016/j.microc.2009.10.016.

(7) Arman, A.; Sağlam, Ş.; Üzer, A.; Apak, R. Direct Electrochemical Determination of Peroxide-Type Explosives Using Well-Dispersed Multi-Walled Carbon Nanotubes/Polyethyleneimine-Modified Glassy Carbon Electrodes. *Anal. Chem.* **2021**, *93* (33), 11451–11460. https://doi.org/10.1021/ACS.ANALCHEM.1C01397.
